# Towards a minimal generic set of domains of functioning and health

**DOI:** 10.1186/1471-2458-14-218

**Published:** 2014-03-03

**Authors:** Alarcos Cieza, Cornelia Oberhauser, Jerome Bickenbach, Somnath Chatterji, Gerold Stucki

**Affiliations:** 1 Faculty of Social and Human Sciences, School of Psychology, University of Southampton, Highfield Campus, SO17 1BJ Southampton, UK; 2 Department of Medical Informatics, Biometry and Epidemiology – IBE, Public Health and Health Services Research, Research Unit for Biopsychosocial Health, Ludwig-Maximilians-University (LMU), Munich, Germany; 3 Swiss Paraplegic Research, Nottwil, Switzerland; 4 ICF Research Branch in cooperation with the WHO Collaborating Centre for the Family of International Classifications in Germany (at DIMDI), Cologne, Germany; 5 Department of Measurement and Health Information Systems, World Health Organization, Multi-Country Studies, Geneva, Switzerland; 6 Department of Health Sciences and Health Policy, University of Lucerne, Lucerne, Switzerland

**Keywords:** ICF, Functioning, Health, Data comparability

## Abstract

**Background:**

The World Health Organization (WHO) has argued that functioning, and, more concretely, functioning domains constitute the operationalization that best captures our intuitive notion of health. Functioning is, therefore, a major public-health goal. A great deal of data about functioning is already available. Nonetheless, it is not possible to compare and optimally utilize this information. One potential approach to address this challenge is to propose a *generic and minimal set* of functioning domains that captures the experience of individuals and populations with respect to functioning and health. The objective of this investigation was to identify a minimal generic set of ICF domains suitable for describing functioning in adults at both the individual and population levels.

**Methods:**

We performed a psychometric study using data from: 1) the German National Health Interview and Examination Survey 1998, 2) the United States National Health and Nutrition Examination Survey 2007/2008, and 3) the ICF Core Set studies. Random Forests and Group Lasso regression were applied using one self-reported general-health question as a dependent variable. The domains selected were compared to those of the World Health Survey (WHS) developed by the WHO.

**Results:**

Seven domains of the International Classification of Functioning, Disability and Health (ICF) are proposed as a minimal generic set of functioning and health: energy and drive functions, emotional functions, sensation of pain, carrying out daily routine, walking, moving around, and remunerative employment. The WHS domains of self-care, cognition, interpersonal activities, and vision were not included in our selection.

**Conclusions:**

The minimal generic set proposed in this study is the starting point to address one of the most important challenges in health measurement – the comparability of data across studies and countries. It also represents the first step in developing a common metric of health to link information from the general population to information about sub-populations, such as clinical and institutionalized populations.

## Background

Functioning is a universal human experience. Functioning as defined in the World Health Organization (WHO) International Classification of Functioning, Disability and Health (ICF) includes the physiological and psychological functions of body systems, as well as the tasks and actions of individuals and their involvement in all life situations [1]. In this sense, disability is a decrease in functional capacity in one or more domains. The overall level of an individual’s functioning varies over his or her lifetime as a result of the interaction between health condition(s) and environmental and personal factors. Every individual will experience some degree of disability as a result of disease or injury or merely the process of aging [2,3]. The WHO has argued that functioning and functioning domains constitute the operationalization that best captures our intuitive notion of health [4]. A high level of functioning is, therefore, a major public health goal of the WHO.

In *clinical care*, health-care providers use functioning information as the starting point for planning interventions [5] and for tracking the impact of a health condition on a person’s life for clinical outcome assessment to evaluate the beneficial or adverse effects of health interventions [6,7]. Information about functioning is also essential for *health-service planning and health-resource allocation*[8] and is becoming a fundamental requirement for reimbursement, as diagnosis alone does not predict service utilization, and information on functioning is required for an adequate prospective payment system [9]. In some countries, “function-related groups” based on the Functional Independence Measure are being used as reimbursement parameters [10]. For *health policy*, functioning and disability serve as suitable descriptors of population health, and data collected provides the evidence for both policy development and evaluation of the effectiveness of policy initiatives [11]. As a result, the United Nations has recommended that national and international disability statistics be collected using functioning domains [12].

In all three areas, but especially in clinical care and health policy, a great deal of data about functioning is already available. Nonetheless, it is not possible to compare and optimally utilize this information within each area, from one area to another, or, most importantly, to use the information from all three areas for public-health purposes, such as for health-system performance assessment or the development and monitoring of prevention and health-promotion programs.

A potential approach to the issue of comparability lies in the development of a *generic and minimal set* of functioning domains that captures the experience of individuals and populations with respect to functioning and health. Only with a common core of domains will it be possible to develop a common metric of functioning and health that calibrates data from all other contexts or data sources, even when data about domains other than those in the *generic and minimal set* are also included [13]. To say that the set is generic means that it must be applicable to all people, i.e. universally. To say that a set is minimal means that it is a set with the least number of domains of functioning that can explain significant differences among people with different degrees of health.

At the population level, the most advanced proposal for a minimal and generic set of domains to date is the WHO’s selection of domains of functioning for describing health in the World Health Survey (WHS), which provides the basis for health-system performance assessments [11]. These domains are: mobility, self-care, pain and discomfort, cognition, interpersonal activities, vision, sleep and energy, and affect. The development of this set of domains was based on extensive, sophisticated and multi-method studies carried out at the WHO over a five-year period [14,15]. There has not been any other international effort of this magnitude. There have also been no attempts outside of the WHO to investigate the relevance of this set of domains across different populations, and, in particular, in the clinical population.

At the individual or clinical level, the most evidence-based proposal of what to measure in clinical populations is the international collaborative effort to develop ICF Core Sets [16]. ICF Core Sets have already been developed for a wide range of health conditions and service settings. The open question for the ICF Core Set initiative is how to compare functioning across conditions and settings and across the general population.

The objective of this study was to identify a minimal generic set of domains suitable for describing functioning in adults, both at the individual and population levels. Since the ICF is the standard classification for describing functioning at all levels, this study used this classification as a reference for the definition of domains (in the ICF domains are called ‘categories’).

The specific aims were: first, to determine whether the WHS domains are relevant for both clinical populations and the general population; secondly, to determine whether additional domains need to be added to the WHS to facilitate comparability across clinical populations.

## Methods

### Study design and data sources

This is a psychometric study using data from three sources: 1) the German National Health Interview and Examination Survey 1998 (GHS), 2) the United States National Health and Nutrition Examination Survey 2007/2008 (NHANES), and 3) the ICF Core Set studies.

The GHS was the first German Health Survey to include the former East and West Germany together in one survey. It was carried out between 1997 and 1999. The data available for public use include information about 7124 adults from a representative sample of the residential population in Germany [17]. The GHS data are available on demand for scientific purposes from the Robert Koch Institute in Berlin [18].

The NHANES survey was performed by the National Center for Health Statistics (NCHS) of the Centers for Disease Control and Prevention designed to assess the health and nutritional status of adults and children in the United States [19]. The data used for this study are from 6228 persons 18 years and older from the 2007–2008 cohort. NHANES data are openly available from the corresponding webpage [20].

The ICF Core Set studies are a series of 22 studies carried out at the ICF Research Branch of the WHO Collaborating Centre for the Family of International Classifications in Germany from 2004 to 2010 in collaboration with institutions in 44 countries in clinical settings ranging from early post-acute to primary care [21]. In total, the data of 9863 persons were available. Although the ICF Core Sets include ICF categories of all the components of the ICF, only categories of the components of functioning (body functions and structures, activities and participation) were further considered. The data on the ICF Core Set studies is also publicly available upon request from the ICF Research Branch [22].

To make the data from all three sources comparable, the questions contained in the GHS and NHANES databases were linked to the categories of the ICF by established linking rules [23]. For example, the variable “DPQ040 [Over the last 2 weeks, how often have you been bothered by the following problems:] feeling tired or having little energy?” of the NHANES was linked to the ICF category b130 Energy and drive functions. Only the data from those questions that could be unequivocally linked to a single ICF category of the components body functions and structures or activities and participation were further considered. Although a large number of contextual factors (environmental and personal) are included in the three data sources used in this investigation, we did not consider them because our focus is on functioning and health. In this context, this investigation can be seen as one of a series of steps. Once we have determined a minimal generic set of functioning domains, those environmental and personal factors with explanatory power in relation to them can be investigated.

At this stage in the study, 257 variables were available for further steps: 1) 25 questions from the GHS, 2) 28 questions from NHANES, and 3) 204 ICF categories from the ICF Core Set studies.

To make sure that all relevant (and only relevant) variables were included in the analyses, the next step consisted of pre-selecting those variables that reflect the ICF categories addressed in:

1) the questions used in the WHS to address its eight domains,

2) the 17 questions used in the Washington City Group extended set [24],

3) the questions contained in 3 of the 6 most commonly-used health-status measures, and

4) the ICF categories of the dimension ‘functioning’ found in at least 11 of the 22 ICF Core Sets.

The questions in 1) and 2) were linked to the ICF using the same rules as for the GHS and NHANES. The questions in 3) had already been linked to the ICF [25]. For 3) and 4) we used the 50% cut-off (3 out of 6 health-status measures and 11 out of 22 ICF Core Sets). Although any cut-off threshold is, in a sense, arbitrary, we decided to use the 50% threshold because it captures the majority of relevant ICF categories.

Since both variables from each data source and the pre-selection criteria were expressed in the standard ICF language, the selection of variables could be performed using these criteria. This pre-selection resulted in 14 variables from GHS, 20 from NHANES, and 56 from the ICF Core Set studies which were included in the analyses.

### Analysis

Descriptive statistics were used to characterize the study populations of all three data sources in terms of age, gender, and percent of people living alone.

Regression methodologies were applied using the self-reported general-health question common to the GHS, the NHANES, and the ICF Core Set studies -- “In general, would you say your health is (excellent/very good/good/fair/poor)?” -- as a dependent variable. In accordance with previous investigations, the response options were transformed as follows: excellent = 5.0, very good = 4.4, good = 3.4, fair = 2.0, and poor = 1.0 [26].

Two regression methodologies were applied for the sake of robustness – Random Forests and Group Lasso regression [27-30]. Both were applied to the data from the GHS, the NHANES, and the ICF Core Set studies. The analyses were also carried out for each data set separately for the ICF categories contained in the ICF components ‘body functions and structures’ and ‘activities and participation’, which were used as independent variables.

Random Forests is a non-parametric regression technique that can be used to rank independent variables according to their level of explanatory relevance based on a so-called variable-importance measure assigned to each independent variable [31]. Group Lasso regression is a parametric regression technique that allows for the selection of the ordinal independent variables that explain most of the variance of a dependent variable by taking their ordinal structure into account. Group Lasso can be used to rank independent variables according to their level of explanatory relevance based on the maximal size of the penalty for which the variable is first selected into the model [32,33]. ICF categories are designated as relevant independent variables when their ranks resulting from Random Forests and Group Lasso regressions are among the top 50% in both regression methodologies. In other words, those ICF categories with the best and most robust predictive value in both regression methodologies are considered relevant.

We considered a WHS domain was valid for both the general and clinical populations when ICF categories addressing this domain were above the 50% cut-off in both clinical and general populations.

We determined that an ICF category needed to be added to the WHS domains when functioning and health are assessed in clinical populations if the ICF category was above the 50% cut-off in the clinical population.

The descriptive statistics, the Random Forests, and the Group Lasso regressions, were performed with R version 2.11.1 [34].

## Results

The number of cases for which the dependent variable was available was 6224 in the GHS, 4436 in the NHANES, and 9264 in the ICF Core Set studies.

The age, gender, and percentage of persons living alone in all three samples are presented in Table 1. The variables from the GHS, NHANES, and ICF Core Set studies are listed as ICF categories and organized according to the components of the ICF in Tables 2 and 3 across the three data sets. The respective ranking obtained from the two regression techniques is also shown. The ICF categories most associated with the self-report of health are those with the highest ranks (i.e. the lowest numbers) across the different data sets.

**Table 1:** Demographics of the study populations of the three datasets used for the regression analyses

	**GHS (n = 6224)**	**NHANES (n = 4436)**	**ICF Core Set studies (n = 9264)**
**Males %**	48.6	48.8	44.6
**Age: years mean (sd)**	45.8 (15.9)	48.5 (17.3)	53.1 (15.9)
**Living alone %**	29.6	12.5	18.7

**Table 2:** Rank order of ICF ‘body functions’ categories

**ICF code**	**Title**	GHS*		NHANES*		ICF Core Set studies*	
		**Random Forests**	**Group Lasso**	**Random Forests**	**Group Lasso**	**Random Forests**	**Group Lasso**
b126	Temperament and personality functions	8	8			13	**9.5**
**b130**	**Energy and drive functions**	**2**	**3**	**2**	**1**	**6**	**4.5**
**b134**	**Sleep functions**	7	**5**	4	**2**	**3**	**2**
b140	Attention functions			6	6	15	17
b144	Memory functions			5	5	17	19
**b152**	**Emotional functions**	**4**	6	**3**	**3**	**5**	**6**
b180	Experience of self and time functions					19	15.5
**b210**	**Seeing functions**	**5**	**4**			16	14
b230	Hearing functions	6	7	**1**	4	18	18
**b280**	**Sensation of pain**	**1**	**1**			**1**	**1**
**b455**	**Exercise tolerance functions**					**2**	**4.5**
b530	Weight maintenance functions	9	9			11	11
**b640**	**Sexual functions**					**7**	**8**
**b710**	**Mobility of joint functions**					**8**	**7**
**b730**	**Muscle power functions**					**4**	**3**
b740	Muscle endurance functions					**10**	15.5
**b780**	**Sensations related to muscles and movement functions**	**3**	**2**			**9**	12
s750	Structure of lower extremity					14	13
s760	Structure of trunk					12	**9.5**
Cut-off point (top 50% of ranking)		5	5	3	3	10	10

List of ICF ‘Body functions’ categories from the GHS, the NHANES, and the ICF Core Set studies datasets included in the analyses, rank order from Random Forests and Group Lasso indicating the level of association with the general health question, and cut-off rank for the different datasets. Those categories that rank among the top 50% in both regression methodologies in at least one dataset were considered confirmed and selected for comparison with the World Health Survey domains of functioning.

*The ICF categories containing a rank number in these columns were included in the analyses with data of this study. In each column, bold numbers indicate that the corresponding ICF category ranked among the top 50% in the corresponding regression methodology for the selected dataset.

**Table 3:** Rank order of ICF ‘activities and participation’ categories

**ICF code**	**Title**	GHS*		NHANES*		ICF Core Set studies*	
		**Random Forests**	**Group Lasso**	**Random Forests**	**Group Lasso**	**Random Forests**	**Group Lasso**
**d110**	**Watching**			**1**	**2**	36	35.5
d115	Listening					37	35.5
d160	Focusing attention					33	31
d175	Solving problems					31	**15.5**
**d230**	**Carrying out daily routine**	**1**	**1**			**14**	**18**
**d240**	**Handling stress and other psychological demands**					**3**	**7**
d310	Communicating with - receiving - spoken messages					30	19.5
d335	Producing nonverbal messages					35	35.5
**d410**	**Changing basic body position**	**2**	**3**	**7**	**5**	**16**	31
**d415**	**Maintaining a body position**			**4**	**3**	23	31
d430	Lifting and carrying objects	4	5	**5**	8	**19**	19.5
d440	Fine hand use			9	12	28	22
**d445**	**Hand and arm use**			**6**	**4**	27	22
**d450**	**Walking**	5	4	**3**	**6**	**8**	**5**
**d455**	**Moving around**	**3**	**2**	11	9	**6**	**3**
d465	Moving around using equipment					29	25.5
**d470**	**Using transportation**					**13**	**12**
d475	Driving					33	**13.5**
**d510**	**Washing oneself**					**2**	**4**
d520	Caring for body parts					20	35.5
d530	Toileting					25	31
**d540**	**Dressing**			12	11	**5**	**6**
d550	Eating			14	13.5	26	27.5
**d570**	**Looking after one’s health**					**11**	**9**
d620	Acquisition of goods and services					22	24
d630	Preparing meals			13	13.5	**18**	27.5
**d640**	**Doing housework**			10	10	**4**	**2**
**d660**	**Assisting others**					**8**	**8**
**d710**	**Basic interpersonal interactions**					**10**	**17**
d760	Family relationships					21	**13.5**
**d770**	**Intimate relationships**					**12**	**10**
d830	Higher education					32	25.5
d845	Acquiring, keeping and terminating a job					**17**	22
**d850**	**Remunerative employment**			**2**	**1**	**15**	**11**
d870	Economic self-sufficiency					24	**15.5**
d910	Community life					**7**	31
**d920**	**Recreation and leisure**			8	**7**	**1**	**1**
Cut-off point (top 50% of ranking)		3	3	7	7	19	19

List of ICF ‘Activities and participation’ categories from the GHS, the NHANES, and the ICF Core Set studies datasets included in the analyses, rank order from Random Forests and Group Lasso indicating the level of association with the general health question, and cut-off rank for the different datasets. Those categories that rank among the top 50% in both regression methodologies in at least one dataset were considered confirmed and selected for comparison with the World Health Survey domains of functioning.

*The ICF categories containing a rank number in these columns were included in the analyses with data of this study. In each column, bold numbers indicate that the corresponding ICF category ranked among the top 50% in the corresponding regression methodology for the selected dataset.

Based on the criterion that ICF categories are designated as relevant independent variables when they rank among the top 50% in both regression methodologies, 10 ICF ‘body functions’ and 18 ‘activity and participation’ ICF categories were identified as most associated with self-reported general health.

Table 4 arranges the final data into three sections according to the first and second specific aims of this study. Each section is arranged by the 8 WHS domains of functioning linked with the specific ICF categories that are above the 50% relevance cut-off in the three data sets.

**Table 4:** Comparison of results with WHS domains - WHS domains of functioning and ICF categories found explanatory for self-perceived health

**WHS domains of functioning**	**Specific ICF categories**		**GHS**	**NHANES**	**ICF Core Set studies**
	**ICF code**	**Title**			
Section A: ICF categories found explanatory for self-perceived health both in the general and clinical population studies					
**Mobility**	d450	Walking	-	✓	✓
	d455	Moving around	✓	-	✓
Self care					
**Pain and discomfort**	b280	Sensation of pain	✓		✓
Cognition					
Interpersonal activities					
Vision					
**Sleep and energy**	b130	Energy and drive functions	✓	✓	✓
**Affect**	b152	Emotional functions	-	✓	✓
*Non-WHS domains*	d230	Carrying out daily routine	✓		✓
	d850	Remunerative employment		✓	✓
Section B: ICF categories found explanatory for self-perceived health only in the general population studies					
**Mobility**	b780	Sensations related to muscles and movement functions	✓		-
	d410	Changing basic body position	✓	✓	-
	d415	Maintaining a body position		✓	-
	d445	Hand and arm use		✓	-
Self care					
Pain and discomfort					
Cognition					
Interpersonal activities					
**Vision**	b210	Seeing functions	✓		-
	d110	Watching		✓	-
Sleep and energy					
Affect					
Section C: ICF categories found explanatory for self-perceived health only in the clinical population studies					
**Mobility**	b455	Exercise tolerance functions			✓
	b710	Mobility of joint functions			✓
	b730	Muscle power functions			✓
	d470	Using transportation			✓
**Self care**	d510	Washing oneself			✓
	d540	Dressing		-	✓
	d570	Looking after one’s health			✓
Pain and discomfort					
Cognition					
**Interpersonal activities**	d710	Basic interpersonal interactions			✓
	d920	Recreation and leisure		-	✓
Vision					
**Sleep and energy**	b134	Sleep functions	-	-	✓
Affect					
*Non-WHS domains*	b640	Sexual functions			✓
	d770	Intimate relationships			✓
	d240	Handling stress and other psychological demands			✓
	d640	Doing housework		-	✓
	d660	Assisting others			✓

Legend: ✓ means that data on the ICF category were available and the ICF category was confirmed for the corresponding dataset as its ranks resulting from Random Forests and Group Lasso regression were among the top 50% in both regression methodologies. - means that data on the ICF category were available, but it was not confirmed based on the 50% cut-off criterion for the corresponding dataset. Space means that no data on the ICF category were available for the corresponding dataset. Empty lines mean that no ICF category could be confirmed by the corresponding combination of datasets for the corresponding WHS domain.

WHS: World Health Survey; ICF: International Classification of Functioning, Disability and Health; GHS: German National Health Interview and Examination Survey 1998; NHANES: United States National Health and Nutrition Examination Survey 2007/2008.

Section A of Table 4 shows which WHS domains are considered valid for both the clinical and the general populations (‘Mobility’, ‘Pain and discomfort’, ‘Sleep and energy’, and ‘Affect’). The table also shows the specific ICF categories that confirm those WHS domains: d450 Walking, d455 Moving around, b280 Sensation of pain, b130 Energy and drive functions, and b152 Emotional functions.

Section A of Table 4 also shows that d230 Carrying out daily routine and d850 Remunerative employment are relevant to self-perceived health in both general and clinical populations. These two additional ICF categories and the five above-mentioned ICF categories represent our recommendation of ICF categories which belong to the minimal generic set of ICF categories suitable for describing functioning both at the individual and population levels.

Section B of Table 4 gives the relevant WHS domains for the general population alone. It shows that the WHS domain ‘Vision’ has been confirmed for the general population based on ICF categories b210 Seeing functions and d110 Watching. It also shows the ICF categories that confirmed the relevance of the WHS domain ‘Mobility’ for the general population alone.

Section C of Table 4 gives the relevant WHS domains for the clinical population alone. It shows the ICF categories that confirmed the WHS domains ‘Self-care’ and ‘Interpersonal activities’. The ICF categories that confirm the relevance of the WHS domain ‘Mobility’ and ‘Sleep and energy’ for the clinical population are also presented. Five ICF categories have also been identified as relevant to self-perceived health in the clinical population: b640 Sexual functions, d770 Intimate relationships, d240 Handling stress and other psychological demands, d640 Doing housework, and d660 Assisting others.

## Discussion

This study proposed the following set of ICF categories as a minimal generic set of functioning and health:

b130 Energy and drive functions

b152 Emotional functions

b280 Sensation of pain

d230 Carrying out daily routine

d450 Walking

d455 Moving around

d850 Remunerative employment

Based on the criteria of relevance used in this study, 4 of the 8 WHS domains of functioning were found to be sufficiently explanatory for self-perceived health in the general and clinical populations. The other WHS domains not represented in the proposed minimal generic set are ‘Vision’, which was only confirmed with data from the general population, ‘Self-care’, and ‘Interpersonal activities’, which were only confirmed with data from the clinical population, and ‘Cognition’, which could not be confirmed at all.

The construction of a minimal generic set requires difficult decisions, and there will always be good reasons for and against each proposed ICF category. Excluding any category must not be interpreted as claiming the category is irrelevant. The minimal generic set exercise, however, demands fine distinctions among relevant categories, and, as long as evidence can be provided for the decision, one can have confidence in the result. In this study, we provide statistical evidence involving large clinical and general population samples. Since in our selection d230 Carrying out daily routine was included, it is not surprising that some WHS domains were not included. These domains are an integral part of people’s lives, but are not as strongly correlated to self-perceived general health.

The proposed minimal generic set of ICF categories of functioning and health can always be augmented for specific applications. This study provides some evidence for the decision about what other ICF categories to add. As can be seen in Table 4, additional mobility ICF categories can be included in the general population studies. The inclusion of ICF categories for vision or watching is also recommended.

The minimal generic set can be operationalized with self-report questions for use in surveys. For the 4 WHS domains of ‘Mobility’, ‘Pain and discomfort’, ‘Sleep and energy’, and ‘Affect’, the WHS itself provides public-domain questions that have been extensively psychometrically studied and widely used around the world [35,36]. The potential users of the WHS questions have to be aware that the domain ‘Mobility’ in the WHS includes both ICF categories on ‘Body functions’ and ‘Activities and participation’. For the operationalization of the two additional categories, d230 Carrying out daily routine and d850 Remunerative employment, there are also good candidate questions from the many widely-used health-status measures that have already been linked to the ICF [37].

Countries can also take advantage of the results of this study when designing a disability survey. Section C of Table 4 presents those ICF categories which are exclusively relevant to persons with health conditions who experience disability or who are at risk of becoming disabled. Disability surveys usually target these persons with the objective of describing their problems or their needs in different areas of life. It is always difficult to decide which relevant domains will help to achieve this objective. A recent comparison of over 100 disability surveys showed that, despite some attempts at harmonization [24], disability surveys are extremely diverse in the domains they address [38]. The set of ICF categories presented in Section C of Table 4 can be seen as a proposal of ICF categories that reliably describe disability. This proposal has been taken into account in a current project conducted by the WHO and the World Bank to develop a Model Disability Survey. All those categories of the Generic Set, as well as those that might be called the “disability set”, are captured in the model disability survey.

There are several limitations to this study. Our general population data came from high-resource Western countries, which do not represent the general worldwide population. This fact led to the choice of ‘remunerative employment’ rather than the more general term ‘work’. The data comes from the adult, non-institutionalized population and might have been different if children and institutionalized populations had been included. Data from many questions and ICF categories came exclusively from clinical populations rather than the general population. We cannot be sure that the same ICF categories would have been found as highly explanatory for both the general and clinical populations if we had included more general-population data. Relying on the self-reported general-health question as the only dependent variable may also be a limitation, since this question may not be suitable to monitor population health trends over time [39]. Nonetheless, self-rated general-health questions have been shown to be strong predictors of functioning and disability and are sensitive to the full spectrum of health conditions [40].

The WHO group responsible for the selection of the WHS domains proceeded according to 5 criteria [14]: These domains must be: 1) valid in terms of intuitive, clinical, and epidemiological concepts of health; 2) linked to the conceptual framework of the ICF; 3) amenable to self-report, observation, or direct measurement; 4) sufficiently comprehensive to capture all important aspects of health states that people value; and 5) comparable across populations. We were guided by these criteria as well. We believe that the 7 ICF categories in our proposed minimal generic set satisfy the first three criteria. We recognize that the next essential step for future research is to identify the extent to which these ICF categories satisfy the last two criteria, namely capturing the aspects of health that people value and being comparable across populations. These two criteria are essential for the next and most important challenge yet to be resolved in health assessment, i.e. to develop a common metric of health to link information from the general population to information about sub-populations, such as clinical and institutionalized populations. Such a metric would be useful for assessing and comparing levels and patterns in the functional course of a person’s life and, thus, trends in population health.

## Conclusions

The minimal generic set proposed in this study is the starting point to address one of the most important challenges in health measurement, namely the comparability of data across studies and countries. It also represents the first step in developing a common metric of health to link information from the general population to information about sub-populations, such as clinical and institutional populations.

## Competing interests

The authors declare that they have no competing interest.

## Authors’ contributions

AC, JB and GS conceived and supervised the study and drafted the manuscript. CO carried out the statistical analysis. SC critically revised the manuscript in several drafting rounds. All authors read and approved the final manuscript.

## Authors’ information

This paper is part of a PhD thesis written by the second author at the medical faculty of Ludwig-Maximilians-University in Munich, Germany.

## Pre-publication history

The pre-publication history for this paper can be accessed here:

http://www.biomedcentral.com/1471-2458/14/218/prepub
